# Decentralized Sensor Fault-Tolerant Control of DC Microgrids Using the Attracting Ellipsoid Method

**DOI:** 10.3390/s23167160

**Published:** 2023-08-14

**Authors:** Hisham M. Soliman, Ehab H. E. Bayoumi, Farag A. El-Sheikhi, Michele De Santis

**Affiliations:** 1 Department of Electrical Power Engineering, Faculty of Engineering, Cairo University, Cairo 11562, Egypt; mohammedhisham@eng.cu.edu.eg; 2 Department of Mechanical Engineering, Faculty of Engineering, The British University in Egypt, El Sherouk City, Cairo 11837, Egypt; ehab.bayoumi@bue.edu.eg; 3 Department of Electrical and Electronics Engineering, Istanbul Esenyurt University, Istanbul 07800, Turkey; farag.elsheikhi@gmail.com; 4 Department of engineering, University Niccolò Cusano, 00166 Roma, Italy

**Keywords:** sensor failure, fault-tolerant control, DC microgrids, attracting ellipsoid method

## Abstract

System stability deterioration in microgrids commonly occurs due to unpredictable faults and equipment malfunctions. Recently, robust control techniques have been used in microgrid systems to address these difficulties. In this paper, for DC-islanded microgrids that have sensors faults, a new passive fault-tolerant control strategy is developed. The suggested approach can be used to maintain system stability in the presence of flaws, such as faulty actuators and sensors, as well as component failures. The suggested control is effective when the fault is never recognized (or when the fault is not being precisely known, and some ambiguity in the fault may be interpreted as uncertainty in the system’s dynamics following the fault). The design is built around a derived sufficient condition in the context of linear matrix inequalities (LMIs) and the attractive ellipsoid technique. The ellipsoidal stabilization idea is to bring the state trajectories into a small region including the origin (an ellipsoid with minimum volume) and the trajectories will not leave the ellipsoid for the future time. Finally, computational studies on a DC microgrid system are carried out to assess the effectiveness of the proposed fault-tolerant control approach. When compared with previous studies, the simulation results demonstrate that the proposed control technique can significantly enhance the reliability and efficiency of DC microgrid systems.

## 1. Introduction

Fault-Tolerant Control (FTC) is concerned with systems whose normal operation is disrupted by a failure in actuators, sensors, or other system components. A component’s malfunctioning can be complete, referred to as a failure [1,2,3], or partial, referred to as fault [4]. This paper focuses on sensor faults.

Passive Fault-Tolerant Control (PFTC) or Active Fault-Tolerant Control (AFTC) can be used to achieve FTC [4]. In the first case, a single controller is designed to stabilize the system and provide the required performance regardless of the failure. This controller does not need to be aware of the fault. AFTC, on the other hand, reconfigures the controller by identifying and isolating the system fault. Many approaches have addressed fault detection and isolation [5].

For example, the sliding-mode observers (SMOs) effectively handle the nonlinear switch discontinuous term for fault detection and estimation. In linear and nonlinear systems with progressive faults, they can handle disturbances, parametric fluctuations, uncertainties, and unmodeled dynamics. It compensates for observer incompatibilities and preserves system stability and reachability in a limited period [6]. Since uncertainties are inherently present, the fault reconstruction technique for matched faults and uncertainties in [7] does not apply to a broad range of actual systems.

Furthermore, they calculated SMO improvements by practically tweaking LMIs according to Lyapunov stability requirements. It lessens the impact of H-∞ criteria, improves fault estimates, and substantiates efforts to address mismatched uncertainty and defects. This study mathematically determined the period the sliding-mode observer would be available and proved its stability. In [8], H-∞ FTC for actuator and sensor faults in wind energy systems is provided. It makes use of Linear-Quadratic Regulator (LQR)-based state feedback control to mimic variable wind speeds generated by stochastic affine models. Event (fault) monitoring for smart grids is provided in [9]. 

Summarizing: the drawback of AFTC is that it produces a more complex controller. Another disadvantage of AFTC is that fault identification is a challenging task, and in the presence of external disturbances, fault detection algorithms cannot ensure reliable detection. Despite these disadvantages, AFTC has advanced controllers that achieve better performance than PFTC. A PFTC can be expressed as a robust control problem if the sensor’s performance is bounded. This method is the focus of this paper. 

More renewable energy sources have been integrated into the present power systems as electricity consumption has increased rapidly. This trend has increased the appeal of microgrids (MGs), which provide an effective technique for combining a variety of energy resources (e.g., wind and solar energies) [10,11,12]. MGs are small-scale power networks comprised of distributed generation (DG), devices for storage, and loads. DC MGs have received increased attention in recent years as a result of the growing use of DC renewables and loads [13,14,15]. The fundamental goals of DC MG control can be described as voltage regulation, stability, and current sharing. In modern DC MGs, hierarchical control structures are often used to meet these control objectives [16,17,18]. 

Control techniques have progressed from (1) centralized where, one controller is used to stabilize the whole system; but requires measuring all the states; (2) decentralized control, where a controller is installed for each DG and requires only the local information of its DG scheme; to (3) distributed schemes, where the controller for each DG requires local and neighboring DGs information [19,20]. It should be noted that the decentralized control is the most reliable option because it does not require the pricey communication network and time delay that centralized control does. Unlike the distributed control which requires communication with neighbor agents, the decentralized control lacks communication. So, this paper focuses on decentralized control. 

To improve performance, some hierarchical control techniques have been introduced. In order to track the local voltage reference of DC MGs under plug-and-play (PnP) MG operation, decentralized control method has been presented in [20]. Accurate current sharing has been achieved in [21] by using a secondary consensus-based controller. The typical hierarchical control system, however, has its own limiting conditions because of the complexity of two-layer control of DC MGs [22,23]. 

Therefore, recent studies [24,25] have considered the constrained communication channel bandwidth, network packet loss, outside interference, and a noisy atmosphere. A distributed secondary cooperative control strategy with adaptive event-triggering communication has been presented in [26]. With reduced communication effort, typical voltage regulation, and appropriate load distribution are achieved. The issue brought on by considering the uncertainty of power loads in DC MGs has been discussed in [27]. To attain satisfactory performance, the authors provided a technique for distributed secondary H∞ consensus. A sliding mode robust controller is incorporated in [28], which studies the impacts of stochastic behavior. 

In this paper, a new passive fault-tolerant control strategy is developed for DC-islanded MGs that have voltage sensors’ faults. The suggested approach can be used to maintain system stability in the presence of flaws, such as faulty actuators and sensors, as well as component failures. The fault can be unknown to the control system and this ambiguity can be translated as an uncertainty in the dynamics of the system following the fault. The proposed design solves the problem as a robust control problem via a new sufficient linear matrix inequalities (LMI) condition. The invariant ellipsoid method is used to tackle the uncertainty in sensor faults. Finally, the computational results are focused on a DC MG system and are carried out to assess the effectiveness of the proposed fault-tolerant control approach. Compared with previous studies, the simulation results demonstrate that the proposed control technique can significantly enhance the reliability and efficiency of DC MG systems.

The main contributions of this paper are as follows:

(1) A new decentralized voltage tracker design is introduced. The new design technique is based on the Attracting Ellipsoid Approach that is a powerful technique in the robust control theory.
(2) A decentralized state feedback with an integral control is proposed using the current and voltage magnitude of each DG which are the DG states.
(3) To obtain the desired voltage reference tracking performance, it is proposed to use an augmented state feedback controller. Analyzing system stability demonstrates that the suggested controller tolerates sensor faults.
(4) Unlike the difficulties in active fault control (detection and fault evaluation), the proposed robust control is much simpler (one controller), easy to implement, and can cope with sensor fault which is never detected or partially known.

The remainder of this paper is organized as follows: Modeling of DC MG dynamics is briefly discussed in Section 2. Additionally, Section 2 contains the sensor fault model as well as a few more preliminary calculations. Section 3 details the design and analysis of the proposed state feedback with integral control for voltage regulation. In Section 3, the proposed control scheme simulation as well as a comparison with previous published research are provided. Simulation validation of the proposed FTC scheme are detailed in Section 4. Conclusions are provided in Section 5.

## 2. Problem Formulation and System Modeling

**Figure 1:**
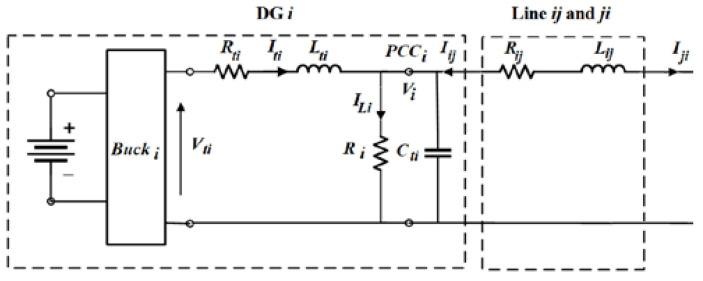
The electrical structure of DG-i.

A DC MG comprised of N DGs connected by DC lines is investigated in this paper. Figure 1 depicts the electrical structure of DG-i.

Each DG has a DC voltage source, a converter, an RLC filter, and a resistive load *R_Li_* as shown in Figure 1. It should be noted that the DGs reliability can be affected by the stochastic and intermittent nature of renewable DC energy sources. In practice, there are several options for energy storage systems to solve this problem. In these instances, the sources might be roughly be operated in a steady-state mode. Renewable energy sources are inherently intermittent. In this study, we assume that PVs include a battery storage system to maintain the output voltage constant.

The dynamic model of DG-i is constructed using Kirchhoff’s voltage and current laws as follows:

(1) $$\left\{\begin{array}{c} \frac{dV_{i}}{dt}=\frac{1}{C_{ti}}\;I_{ti}-\frac{1}{C_{ti}}\;I_{Li}+\frac{1}{C_{ti}}\;I_{ij}\\ \frac{dI_{ti}}{dt}=-\frac{1}{L_{ti}}\;V_{i}-\frac{R_{ti}}{L_{ti}}\;I_{ti}+\frac{1}{L_{ti}}\;V_{ti}\\ Line\;ij:\;\frac{dI_{ij}}{dt}=-\frac{R_{ij}}{L_{ij}}\;I_{ij}+\frac{1}{L_{ij}}\;V_{j}-\frac{1}{L_{ij}}\;V_{i} \end{array}\right.$$

where *V_i_* and *I_ti_* denote the DG-i capacitor voltage and output current, respectively. The command to the converter is represented by *V_ti_*, *R_ti_*, *L_ti_*, and *C_ti_* are constants that represent the filter’s electrical properties. *R_ij_* and *L_ij_* are the power line impedances connecting DG-i and DG-j. Each DG-j’s capacitor voltage is represented by *V_j_*.

It is assumed in (1) that the power lines connecting the DGs possess quasi-stationary dynamics [20], i.e., *dI_ij_*/*dt* = 0. This assumption is valid as the line inductance *L_ij_* in DC systems is significantly small and thereby the line dynamics can be neglected.

(2) $$\left\{\begin{array}{c} ∴\;I_{ij}=\frac{V_{j}-V_{i}}{R_{ij}}\\ \frac{dI_{ti}}{dt}=-\frac{1\;}{L_{ti\;}}V_{i}-\frac{R_{ti}}{L_{ti}}\;I_{ti}+\frac{1}{L_{ti\;}}\;V_{ti}\\ \frac{dV_{i}}{dt}=\frac{1}{C_{ti}}I_{ti}-\frac{1}{C_{ti}}\;I_{Li}+\frac{1}{C_{ti}R_{ij}}V_{j}-\frac{1}{C_{ti}{R_{i}}_{j}}\;V_{i}\; \end{array}\right.$$

The islanded DC MG, shown in Figure 1, contains N DGs that can be modeled through the following state-space equations in the same manner:

(3) $${\dot{x}}_{i}=A_{ii}x_{i}+B_{i}u_{i}+D_{i}w_{i},\;y_{i}=C_{i}x_{i}=z_{i}$$

where $x_{i}={\left[V_{i}\;I_{ti\;}\right]}^{\prime },u_{i}=V_{ti},\;y_{i}=z_{i}$ are states vectors, input, *y_i_* as the measurable outputs, and assume that *y_i_ = x_i_*. The output vector to be optimized is *z_i_*. The matrices *A_ij_*, *B_i_*, are as follows:

(4) $$\left\{\begin{array}{c} A_{ii}=\left[\begin{array}{cc} -\frac{1}{C_{ti}}\sum_{j}\frac{1}{R_{ij}}-\frac{1}{R_{i}C_{ti}} & \frac{1}{C_{ti}}\\ -\frac{1}{L_{ti}} & -\frac{R_{ti}}{L_{ti}} \end{array}\right]\\ A_{ij}=\left[\begin{array}{cc} \frac{1}{R_{ij}C_{ti}} & 0\\ 0 & 0 \end{array}\right]\\ B_{i}=\left[\begin{array}{c} 0\\ \frac{1}{L_{ti}} \end{array}\right]\\ C_{i}=\left[\begin{array}{cc} 1 & 0\\ 0 & 1 \end{array}\right] \end{array}\right.$$

The external disturbance is:

(5) $$D_{i}=[A_{i1}\ldots O_{ii}\ldots A_{iN}],$$

**Figure 2:**
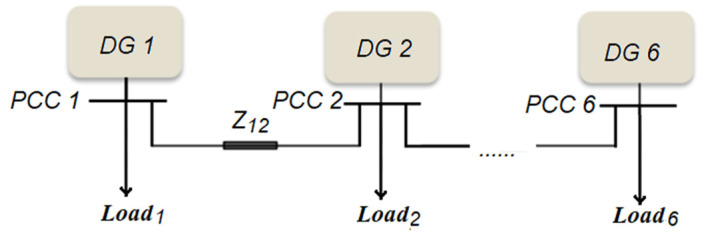
A DC microgrid with six islanded DGs.

The system studied is chosen to be radial as most of the distribution networks are radial. As illustrated in Figure 2, an islanded DC MG case-study system consists of six DGs.

Table 1 and Table 2 outline the electrical parameters of each distributed generation as well as the distribution lines.

The MG study system is discretized with sampling time *T_s_* using Table 1 and Table 2. The discrete-time state equation is provided in the Appendix A. 

It should be noted that graph theory can be used to solve non-radial networks, as described in [29].

The overall MG discrete-time model is as follows using (3–5):

(6) $$x(k+1)=Ax(k)+Bu(k),\;y(k)=Cx(k),\;x(0)=x_{0}$$

The vectors *x*, *u*, and *y* are the state, control, and measurement of dimensions *n*, *m*, and *l*, respectively. Assuming all the states are available for state-feedback control, *C* = *I*. The control objective aims to obtain the output tracking the input with a steady-state error of zero. In addition, the controller must be decentralized which uses only local states.

The number of outputs that can track a reference input vector, *y_r_*, cannot be more than the number of control inputs to maintain controllability. Consequently, the output equation for the open-loop system shown in (6) can be rewritten as:

(7) $$y\left(k\right)=Cx\left(k\right)=\left[\begin{array}{c} C_{1}\\ C_{2} \end{array}\right]x\left(k\right)=\left[\begin{array}{c} y_{1}(k)\\ y_{2}(k) \end{array}\right]$$

where $y_{1}\in R^{h},\;h\leq l$ denotes the vector of the outputs required to follow the reference input vector $y_{r}$. It is to be noticed that the controller is called a regulator if the input is constant; otherwise, it is referred to as a tracker. This section describes the design of the system’s decentralized tracker (6). The interconnected system (6) can be subdivided into N subsystems.

With $A=\left\{A_{i,j}\right\},\;and\;B=blockdiagonl\{B_{1},\ldots {,B}_{N}\}$, $C=blockdiagonl\{C_{1},\ldots {,C}_{N}\}$ subsystem #i is provided by:

(8) $$x_{i}\left(k+1\right)=A_{ii}x_{i}\left(k\right)+B_{i}u_{i}\left(k\right)+D_{i}x\left(k\right),{\;D}_{i}=\left[\begin{array}{ccc} A_{i1}\ldots & O_{ii}\ldots & A_{iN} \end{array}\right],{\;y}_{i}(k)=C_{i}x_{i}(k)i=1,..,N$$

The dimensions of $x_{i},\;u_{i}$ are respectively $n_{i},\;m_{i},\;n=\sum_{i=1}^{N}n_{i},\;m=\sum_{i=1}^{N}m_{i}.$

The decentralization of proposed control can be achieved by reducing the impact of an external disturbance, *D_i_ x*(*k*). The dynamics of the remainder of the system on a specific subsystem are represented by Equation (8), where the vector *x* is supposed to be an external bounded disturbance *w*(*k*). The control decentralization can be performed by minimizing the ellipsoid volume, as will be seen in the sequel.

It is worth noting that the MG model (6) does not have an integrator (it is a type 0 plant). As a result, for a step input, a steady-state error will occur. The output voltage must precisely follow the reference voltage with no errors. To achieve tracking task for subsystem *i*, a vector comparator, and an integrator $z_{i}$ are added which fulfill:

(9) $$z_{i}\left(k+1\right)=z_{i}\left(k\right)+T_{s}[y_{ri}\left(k\right)-y_{1i}\left(k\right)]$$

As a result, the augmented state space representation controls the open-loop system of subsystem-*i* is:

(10) $$\left\{\begin{array}{c} {\hat{x}}_{i}\left(k+1\right)={\hat{A}}_{ii}\hat{x}\left(k\right)+{\hat{B}}_{i}u_{i}\left(k\right)+{\hat{D}}_{i}w\left(k\right)+{\hat{I}}_{i}y_{ri}\left(k\right),\;\\ y_{i}\left(k\right)={\hat{C}}_{i}{\hat{x}}_{i}(k),\; \end{array}\right.$$

where ${\hat{x}}_{i}=\left[\begin{array}{c} x_{i}\\ z_{i} \end{array}\right],{\hat{A}}_{ii}=\left[\begin{array}{cc} A_{ii} & O\\ -T_{s}C_{1i} & I_{i} \end{array}\right],\;{\hat{B}}_{i}=\left[\begin{array}{c} B_{i}\\ O \end{array}\right],{\hat{D}}_{i}=\left[\begin{array}{c} D_{i}\\ O \end{array}\right],{\hat{I}}_{i}=\left[\begin{array}{c} O\\ T_{s}I_{i} \end{array}\right],\;{\hat{C}}_{i}=\left[\begin{array}{cc} C_{i} & 0 \end{array}\right]$.

It is required to design the state feedback plus integral control provided by:

(11) $$u_{i}\left(k\right)={\hat{K}}_{i}\left(k\right){\hat{x}}_{i}\left(k\right)=\left[\begin{array}{cc} K_{i} & K_{Ii} \end{array}\right]{\hat{x}}_{i}\left(k\right)$$

Matrix *C* is an identity matrix for this application. 

Now the linear time-invariant dynamical system (10) when subject to sensor faults becomes:

(12) $$\left\{\begin{array}{c} {\hat{x}}_{i}\left(k+1\right)={\hat{A}}_{ii}\hat{x}\left(k\right)+{\hat{B}}_{i}u_{i}\left(k\right)+{\hat{D}}_{i}w\left(k\right)+{\hat{I}}_{i}y_{ri}\left(k\right),\;\\ y_{i}\left(k\right)={\hat{C}}_{i}{\hat{x}}_{i}(k),\\ y_{si}(k)=diag\left[\varphi_{i}\left(k\right)\right]y_{i}(k) \end{array}\right.$$

where vector *y_i_*(*k*) represents the system output, and *y_s_*(*k*) represents the measured output with a sensor fault. The preceding description can be used to model systems with multiplicative faults such as actuator, sensor, and component failures. For modeling the multiplicative faults, the relevant system matrices should be multiplied by the appropriate matrix. 

*φ_j_*(*k*) is the sensor function which represents the remaining function of the associated sensor. For example, if a sensor *φ_j_*(*k*) = 0.8, in which *φ_j_*(*k*) denotes the remaining function of the *j*th sensor, then the sensor is 80% functioning. In other words, *φ_j_*(*k*) = 0 indicates the sensor failure, while *φ_j_*(*k*) = 1 indicates the sensor works properly. A faulted sensor will then be such that 0 < *φ_j_*(*k*) < 1. Consequently, it is a bounded sensor fault.

The control objective in addition to finding a decentralized dynamic tracker for each DG, should also be robust against sensor fault.

## 3. Decentralized Passive Sensor Fault-Tolerant Control

Each DG-i is supplied with the proportional with integral controllers listed below, (11):

(13) $$u_{i}\left(k\right)={\hat{K}}_{i}\left(k\right){\hat{x}}_{i}\left(k\right)=\left[\begin{array}{cc} K_{i} & K_{Ii} \end{array}\right]{\hat{x}}_{i}\left(k\right)$$

where *K_i_* ∈ *R*^1×2^, *K_Ii_* ∈ *R*^1×1^, and controllers, *i* = *1*, *…*, *N*, are decentralized because the $u_{i}$ computation only requires the DG-i state. The proposed system incorporates proportional state feedback and integral control. It is completely decentralized by means of local states. It avoids communication of bordering subsystems states as in the case of distributed control. The proposed design is simple, contrary to centralized control, which has an expensive communication network and associated delay, which reduces system stability. It should be noted that the communication network in a centralized scheme is prone to failure, which can lead to a total collapse of the control system.

The invariant-set approach [30,31], and its reference applications [32,33,34], is an approach used for designing systems controllers that are disrupted by external disturbances (perturbed systems). An invariant-set is one in which if an initial state vector *x*(*0*) begins within it, the trajectory *x*(*k*) will not leave it for the future time *k* > 0. The invariant set is approximated using a bounding ellipsoid because determining it mathematically is difficult. The invariant ellipsoid technique is employed in linear systems to suppress bounded disturbance by decreasing the ellipsoid volume. In [30], the invariant ellipsoid approach is employed to propose a novel strategy for minimizing the effect of external disturbances on linear systems. When the initial state is outside of the ellipsoid, the ellipsoid is a set that contains the origin and attracts the state trajectory. As a result, it is referred to as an attractive ellipsoid. When the state trajectory arrives at the ellipsoid, it does not leave it as time passes. As a result, the ellipsoid is referred to as an invariant ellipsoid. To reduce the effect of external disturbances on the trajectory, the volume of the ellipsoid must be reduced [31]. The goal of the MG voltage control challenge is to develop a controller that allows the output voltage to track the reference voltage. 

The tracker must also be long-lasting in the event of sensor faults. It should also lessen the impact of disruptions on the output voltage. This is known as a disturbance-rejection tracker.

In (10), *w*(*k*) reflects the bounded external disturbances that are subject to the constraint:

||*w*(*k*)||≤1, ∀*k* ≥ 0 (14)

The symbol ‖(.)‖ denotes the vector (.) Euclidean norm. Note that, the external disturbance is L∞-bounded. To optimize *z*, the design goal is also to minimize the influence of disturbance *Dw* on the output. It should be observed that the disturbance constraint (14) has no effect on generality because the matrix *D* can always be scaled to satisfy (14). Note that the normalization in constraint (14) results in simpler LMI condition than if normalization is not carried out. 

Ref. [30] considers the following problem. Given the discrete time system.

(15) $$x\left(k+1\right)=Ax\left(k\right)+Bu\left(k\right)+Dw\left(k\right),\;y\left(k\right)=Cx\left(k\right),\;subject\;to\;\left\|w(k)\right\|\leq 1$$

The pairs (*A*, *B*) and (*A*, *C*) are assumed controllable, and observable respectively. The state feedback controller *u*(*k*) *= Kx*(*k*), which stabilizes (14) and rejects the disturbance *w*(*k*) in an ideal way (in terms of minimizing the bounding ellipsoid trace of the optimized output, $E_{z}=CPC\prime$) is provided by the following theorem [30].

**Notation.**  *The superscript (.)’ represents matrix transposition throughout the paper, R^n^ denotes the n-dimensional Euclidean space and* $R^{n\times m}$* is the set of all* n×m *real matrices. For a symmetric* $P\in R^{n\times n},P>0$ *indicates that it is positive definite. A symmetric matrix*$\left[\begin{array}{cc} Q+Z+Q^{\prime }+Z^{\prime } & R\\ R\prime & P \end{array}\right]\;is\;denoted\;by\;\left[\begin{array}{cc} \left(Q+Z+*\right) & R\\ {}* & P \end{array}\right]$

**Theorem** **1.** *Ref. [30]*

Let *P*, *Y* be a solution of the minimization optimization problem.

minimizetrCPC′

Subject to the constraints:

$$\left[\begin{array}{ccc} -\alpha P & * & *\\ AP+BY & -P & *\\ 0 & D^{\prime } & -\left(1-\alpha \right)I \end{array}\right]\leq 0,\;P>0,\;\alpha >0$$

For some 0 < α < 1. The minimization is carried out with respect to the matrix variables $P=P^{\prime }$, *Y*, and the scalar parameter α.

Moreover, the optimal state controller, stabilizing (15) and rejecting the disturbance, is provided by:

$$K=YP^{-1}$$

Note that the trace function is adopted due to its linearity; being synonymous to the sum of squared semi-axes of the ellipsoid *E_z_*. This latter condition will be employed throughout the text to help reduce the problem of interest to standard semidefinite programs. The multiplicative term αP is the source of nonlinearity in the above theorem. After fixing α, the above matrix equation becomes linear, making it simple to solve with the LMI toolbox. The scalar α is iteratively updated to solve the minimization problem.

Theorem 1 can be used to solve the sensor FTC as follows. After the occurrence of the sensor fault, the matrix *C_i_* in (4) becomes one of the matrices $diag\left[\varphi_{i,j}\left(t\right)\right]C_{i},\;i=1\ldots N,\;j=1,2$.

The effectiveness reduction in the sensors of DG-i is randomly selected as $\varphi_{i,1}=diag\left[0.81\;0.91\right],\;\varphi_{i,2}=diag\left[0.91\;0.63\right]$.

The design of sensor FTC for DG-i can be performed by replacing *C* in Theorem 1, by $diag\left[\varphi_{i,j}\left(t\right)\right]C_{i},\;$*A* by ${\hat{A}}_{i}$, and *B* by ${\hat{B}}_{i}$. We obtain the following theorem.

**Theorem** **2.** *The sensor FTC of DG-i, i = 1, …, N, is obtained by solving the following optimization problem:*

$$minimize\;\{\max_{j=1,2}tr\;\left\{\left(diag\left[\varphi_{i,j}\left(t\right)\right]C_{i}\right)P_{i}{\left(diag\left[\varphi_{i,j}\left(t\right)\right]C_{i}\right)}^{\prime }\right\},\;i=1\ldots N\}.$$

Subject to the constraints:

$$\left[\begin{array}{ccc} -\alpha {\hat{P}}_{i} & * & *\\ {\hat{A}}_{i}{\hat{P}}_{i}+{\hat{B}}_{i}{\hat{Y}}_{i} & -{\hat{P}}_{i} & *\\ 0 & D\prime & -\left(1-\alpha \right)I \end{array}\right]\leq 0,\;{\hat{P}}_{i}>0,\alpha >0$$

Moreover, the optimal sensor FTC is provided by:

$${\hat{K}}_{i}={\hat{Y}}_{i}{{\hat{P}}_{i}}^{-1},\;i=1\ldots N$$

Solving Theorem 2 (using Matlab LMI, yalmip, and sedumi), the proposed tracker is provided in Table 3.

## 4. Simulation Validation

The system shown in Figure 2 is modelled using the Matlab/SimPower Systems Toolbox. Robust stability, required response, and steady-state capabilities have all been achieved as per the IEEE requirements [35].

The performance of the developed controllers is measured during the randomized effectiveness of the sensor signal (sensor fault). The suggested planned controls are tested in four different scenarios on the study system. Each of the four scenarios is played out by randomly selecting a sensor in a certain DG that has a failure level (reduction in the effectiveness of the sensor signal in percentage). 

Table 4 shows the random sensor faults choices applied to random DGs. The introduced design is a proportional state-feedback system with integral control that is totally decentralized by utilizing local states. Therefore, six controllers are designed, and their gains are provided in Table 3. Note that Theorem 2 is only a sufficient condition so the proposed controller is stabilizing the system for faults more severe than the design faults, Table 4. 

To make a comparative study between the proposed technique and other techniques, a completely decentralized auto-tuned control method is used to design six PI controllers for the six DGs provided in Figure 2. The designed gains for the six auto-tuned PI controllers are illustrated in Table 5.

**Remark** **1.** *Summary of the proposed and auto-tuned control algorithms.*

The proposed algorithm:

- For a given scalar α, the matrix equations in Theorem 2 become linear, solves them by the Matlab LMI toolbox.
- Calculates the objective function $\{\max_{j=1,2}tr\left\{(diag\left[\varphi_{i,j}\left(t\right)\right]C_{i}{)P}_{i}{\left(diag\left[\varphi_{i,j}\left(t\right)\right]C_{i}\right)}^{\prime }\right\},\;i=1\ldots N\}.$
- Updates α iteratively till the minimum of the objective function is obtained (the Matlab command fminsearch can be used).

The auto-tuned algorithm:

The method of setting controller gains based on a study system model or data is known as auto-tune PI. It tunes PI gains in a Simulink model using Simulink Control Design^TM^. The auto-tune PI controller operates using a linearization of the study system model. It computes PI controller gains based on the obtained response to balance robustness and performance.

### 4.1. Scenario 1: The Sensor’s Effectiveness Reduction in one DG

#### 4.1.1. Case 1: Sensor Failure in DG_1_

By constructing a random selection approach and picking a problem in one sensor at a random deterioration level at a random time, we were able to choose the sensor in DG_1_ with 80% sensor effectiveness at t = 7 s, as shown in Table 4.

**Figure 3:**
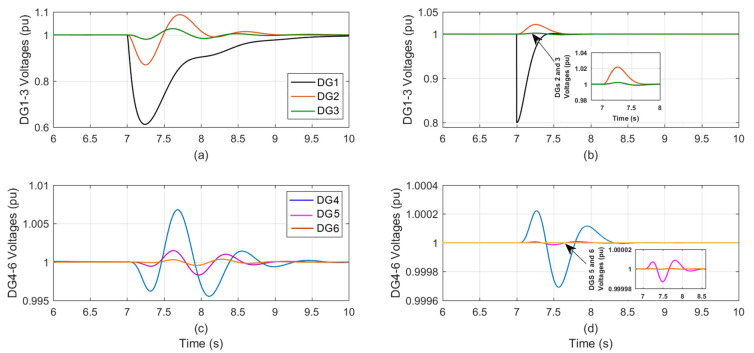
The DGs voltage during sensor effectiveness in DG1 is degraded from 100% to 80%; (**a**,**c**) the conventional PI-tuned; (**b**,**d**) the proposed control.

At t = 7 s, Figure 3 shows the DGs voltage during sensor effectiveness in DG_1_ is degraded from 100% to 80%: Figure 3a,c for the conventional PI auto-tuned technique, and Figure 3b,d for the proposed control technique.

The fault impacts in the other five DGs are significantly severe in the auto-tuned approach while, it is minimal in the proposed one, the effects are close to zero. Table 6 summarizes the implications of 80% sensor effectiveness in DG_1_ on the other five DGs for both control techniques. 

The control parameters shown in Table 6 demonstrate the dead-beat, quick, and zero steady-state performance of the proposed designed trackers for the six DGs and the auto-tuned PI at 80% sensor effectiveness in DG_1_.

#### 4.1.2. Sensor Failure in DG_5_

**Figure 4:**
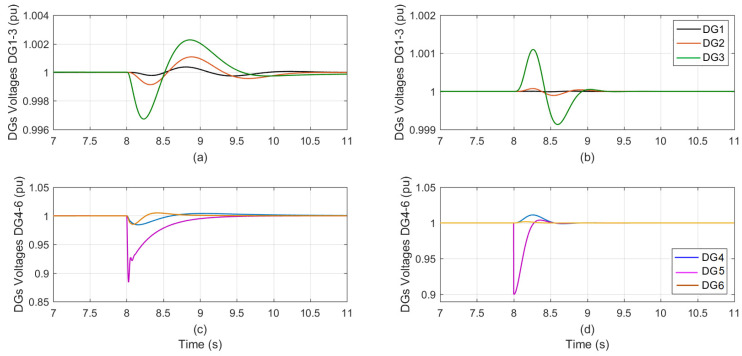
The DGs voltage during sensor effectiveness in DG_5_ is degraded from 100% to 90%; (**a**,**c**) the conventional PI-tuned; (**b**,**d**) the proposed control.

As shown in Table 4, selecting the sensor in DG_5_ with 90% sensor effectiveness at t = 8 s using a random selection technique and a sensor fault at a random degradation level and duration. At t = 8 s the voltage of the DGs is shown in Figure 4a,c for the traditional PI auto-tuned approach, and in Figure 4b,d for the suggested control technique, at a time when the sensor effectiveness in DG5 decreased from 100% to 90%.

The proposed technique has only slight effects on the other five DGs compared with the auto-tuned method, which has a significant impact not only on the faulty sensor DG but also on the remaining five DGs. Table 7 provides a concise summary of the impact that each control technique had on the faulty sensor DG as well as the other five DGs.

### 4.2. Scenario 2: Successive Sensor’s Effectiveness Degrades in Two DGs

The sensor in DG_2_ was randomly picked with 80% effectiveness at t = 8 s while the sensor in DG_4_ was randomly selected with 90% effectiveness at t = 9 s using a randomized selection approach, as shown in Table 4.

**Figure 5:**
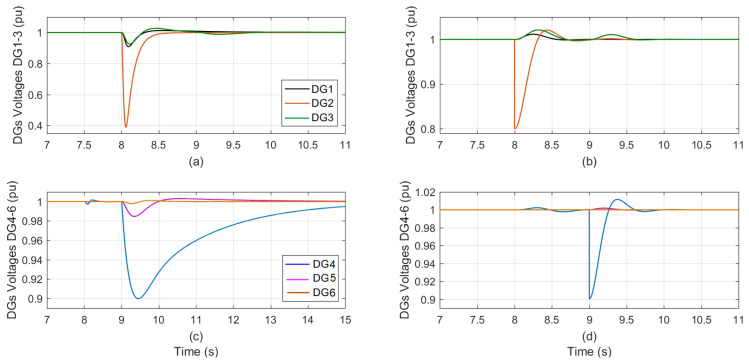
The DGs voltage during successive sensor’s effectiveness degrades in DG_2_ (80%) followed by DG_4_ (90%); (**a**,**c**) the conventional PI-tuned; (**b**,**d**) the proposed control.

Figure 5a,c depict the two successive sensor failures in DG_2_ and DG_4_ when the conventional auto-tuned PI control method is utilized. While Figure 5b,d depict two successive sensor errors in DG_2_ and DG_4_ when the proposed control method is operating.

Since the proposed tracker approaches the fault problem as a disturbance, the fault effects arising in neighboring DG_1_ and DG_3_ during the first fault occurrence in DG_2_ are minimal, as shown in Figure 5b. Moreover, the proposed controls performed admirably during the successive sensor failure in DG_4_ and its neighboring DGs.

Figure 5a,c show the effect on the faulty sensor DGs and their neighboring DGs when the auto-tuned PI controllers are operating.

Table 8 presents a condensed overview of the influence that both control techniques made on the six DGs caused by simultaneous faults in the sensors of DG_2_ and DG_4_.

### 4.3. Scenario 3: Concurrent Sensor’s Effectiveness Degrades in Two DGs inside the Designed Range

Using randomized selection, the sensor in DG_2_ was chosen at random with an effectiveness of 80% at t = 9 s, while the sensor in DG_4_ was selected at random with an effectiveness of 90% at the same time, as shown in Table 4.

**Figure 6:**
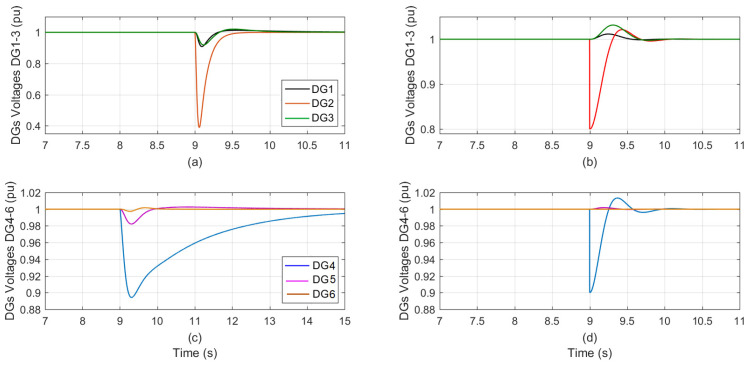
The DGs voltage during concurrent sensor’s effectiveness degrades in DG_2_ (80%) and in DG_4_ (90%) concurrently; (**a**,**c**) the conventional PI-tuned; (**b**,**d**) the proposed control.

When the traditional auto-tuned PI control approach is applied, both DG_2_ and DG_4_ have concurrent sensor failures, which are shown in Figure 6a,c. Figure 6b,d show two different sensor faults happening at the same time in DG_2_ and DG_4_ while the suggested control technique is being applied.

In Figure 6b,d the proposed controllers are tested against this scenario. The results are much better than those for the conventional auto-tune PI controllers provided in Figure 6a,c.

Concurrent faults in the sensors of DG_2_ and DG_4_ are summarized in Table 9, which provides an overview of the impact of both control strategies on all six DGs.

### 4.4. Scenario 4: Concurrent Sensor’s Effectiveness Degrades in Two DGs outside the Designed Range

To put the proposed system through rigorous testing. The suggested system is subjected to two DG sensors operating concurrently with effectiveness values beyond the planned control range.

This was accomplished by picking DG_2_ and DG_4_ at random, with sensor failure effectiveness as shown in Table 4.

**Figure 7:**
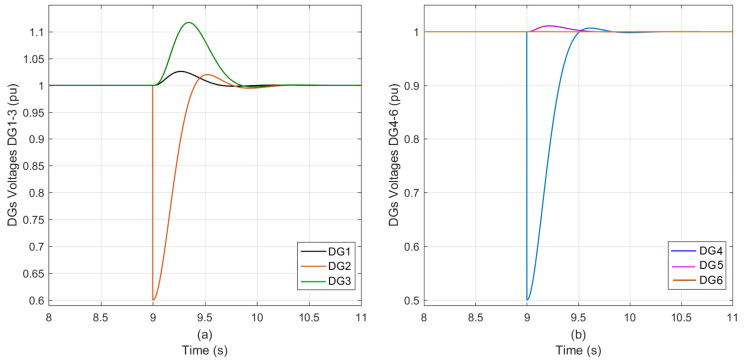
The DGs voltage during concurrent sensor’s effectiveness degrades in DG_2_ (60%) followed by DG_4_ (50%) outside the design range using the proposed control; (**a**) DG_1_, DG_2_, and DG_3_; (**b**) DG_4_, DG_5_, and DG_6_.

The proposed system operated perfectly and rejected the sensors’ disturbance successfully. The proposed trackers respond swiftly and sensor’s fault reflection to the neighboring DGs is minimal, as shown in Figure 7a,b.

The control response parameters for the six DGs are illustrated in Table 10.

## 5. Conclusions

The issue of FTC for DC microgrids is investigated in this paper. The introduced technique for mitigating the impact of sensor faults is a passive FTC scheme. By considering the microgrid under random sensors’ faults, conditions are obtained for PFTC to achieve stability of the closed loop.

These conditions are derived in terms of LMIs for the proposed state feedback with an integral voltage tracker. The results are obtained via modeling the sensors fault as a norm-bounded type parameter uncertainty. The effects of such uncertainties on the system performance are attenuated by minimizing the relevant attracting ellipsoid.

Results from the analysis and simulation studies reveal that the proposed controller has satisfied performance even with the simultaneous faulty sensors and measurements. DC microgrids are susceptible to a variety of failures and faults in practical applications.

Future research should investigate the impact of actuator faults and other types of fault signals on the functioning of DC microgrids. In addition, it should be investigated if the sensor FTC be viewed from another perspective (as if the system is under cyber-attack [36]). Further study is needed in this direction.

## Figures and Tables

**Table 1:** Microgrid parameters [20].

DGs	Parameters of the Buck Converter		Shunt Capacitance *C_t_* (mF)	Load Parameter *R* (Ω)	Power Rating (W)
	R_t_ (Ω)	L_t_ (mH)			
DG_1_	7.22	72.2	25	160	1200
DG_2_	7.22	72.2	32	80	600
DG_3_	7.22	72.2	25	120	900
DG_4_	7.22	72.2	30	160	1200
DG_5_	7.22	72.2	18	100	800
DG_6_	7.22	72.2	12	120	900
*V_dc_* (DC bus voltage) 100 V			*f_sw_* (Switching frequency) 40 kHz		*f_o_* (Nominal frequency) 50 Hz

**Table 2:** Parameters of distribution lines.

Line Impedance (*Z_ij_*)	Line Resistance (*R_ij_*)			Line Inductance (*L_ij_*)		
	*r_ij_* (Ω/m)	Cable Length (m)	*R_ij_* (Ω)	*l_ij_* (μH/m)	Cable Length (m)	*L_ij_* (μH)
*Z*_12_	0.05	180	9	1.8	180	324
*Z*_23_	0.05	240	12	1.8	240	432
*Z*_34_	0.05	300	15	1.8	300	540
*Z*_45_	0.05	240	12	1.8	240	432
*Z*_56_	0.05	264	13.2	1.8	264	475.2

**Table 3:** The proposed tracker.

Controller	α	Ellipsoid Volume	K, K_I_
1	0.05	1.6509 × 10^−16^	[−14.649 −45.078], 42.592
2	0.42	1.639 × 10^−16^	[−59.741 −74.988], 155.51
3	0.03	2.5792 × 10^−16^	[−24.598 −55.922], 22.399
4	0.1	2.6294 × 10^−16^	[−18.248 −43.158], 52.527
5	0.16	2.8791 × 10^−16^	[−98.091 −114.53], 108.91
6	0.42	5.9235 × 10^−16^	[−85.036 −86.043], 145.12

**Table 4:** Reduction in the effectiveness of the sensor signal in percentage.

Scenario Number	Case	DG Number	Time (s)	Effectiveness of the Sensor Signal
1	Case 1	DG_1_	at t = 7 s	[0.8 0.6]
	Case 2	DG_5_	at t = 8 s	[0.9 0.7]
2	One case only	Consecutive faults on DG_2_ and DG_4_	at t = 8 s at t = 9 s	[0.8 0.65] (DG_2_) [0.9 0.75] (DG_4_)
3	One case only	Simultaneous faults on DG_2_ and DG_4_ (within the design control range)	at t = 9 s and at t = 9 s	[0.8 0.65] (DG_2_) [0.9 0.75] (DG_4_)
4	One case only	Simultaneous faults on DG_2_ and DG_4_ (outside the design control range)	at t = 9 s and at t = 9 s	[0.6 0.55] (DG_2_) [0.5 0.35] (DG_4_)

**Table 5:** Auto-tuned PI controllers gains for the six DGs.

PI-Gains	DG_1_	DG_2_	DG_3_	DG_4_	DG_5_	DG_6_
*K_p_*	2.623101	1.554792	2.095479	1.958416	2.518254	2.428056
*K_i_*	50.52526	23.36601	21.52614	21.11258	30.13315	54.55977

**Table 6:** Controller response parameters during sensor effectiveness fault 80% in DG_1_.

		% of Voltage Dip during Sensor Fault (%)		Control Response Parameters					
				% Overshoot (%)		Settling Time (s)		Steady State Error (%)	
		PI-Tuned	Proposed	PI-Tuned	Proposed	PI-Tuned	Proposed	PI-Tuned	Proposed
Output Voltage	DG_1_	39.12	19.35	≈0.0	≈0.0	2.312	0.482	0.012	≈0.0
	DG_2_	12.32	0.252	9.843	2.871	1.834	0.435	≈0.0	≈0.0
	DG_3_	4.781	0.323	2.351	0.335	1.237	0.351	≈0.0	≈0.0
	DG_4_	0.431	0.031	0.734	0.0213	1.051	0.336	≈0.0	≈0.0
	DG_5_	0.219	0.0017	0.204	0.00123	1.047	0.271	≈0.0	≈0.0
	DG_6_	0.078	0.00042	0.197	≈0.0	1.039	0.123	≈0.0	≈0.0

**Table 7:** Controller response parameters during sensor effectiveness fault 90% in DG_5_.

		% of Voltage Dip during Sensor Fault (%)		Control Response Parameters					
				% Overshoot (%)		Settling Time (s)		Steady State Error (%)	
		PI-Tuned	Proposed	PI-Tuned	Proposed	PI-Tuned	Proposed	PI-Tuned	Proposed
Output Voltage	DG_1_	0.043	0.0044	0.047	≈0.0	1.037	0.311	0.0005	≈0.0
	DG_2_	0.106	0.013	0.148	0.035	1.049	0.413	0.0008	≈0.0
	DG_3_	0.237	0.098	0.234	0.124	1.121	0.532	0.0009	≈0.0
	DG_4_	2.232	0.217	1.12	1.45	1.534	0.456	0.047	≈0.0
	DG_5_	12.07	9.841	≈0.0	0.129	1.765	0.512	0.051	≈0.0
	DG_6_	2.354	0.221	1.17	0.892	1.627	0.488	0.0481	≈0.0

**Table 8:** Controller response parameters during simultaneous 80% sensor effectiveness in DG_2_ followed by 90% sensor effectiveness in DG_4_.

		% of Voltage Dip during Sensor Fault (%)		Control Response Parameters					
				% Overshoot (%)		Settling Time (s)		Steady State Error (%)	
		PI-Tuned	Proposed	PI-Tuned	Proposed	PI-Tuned	Proposed	PI-Tuned	Proposed
		DG_2_	DG_4_	DG_2_ DG_4_	DG_2_ DG_4_	DG_2_ DG_4_	DG_2_ DG_4_	DG_2_ DG_4_	DG_2_ DG_4_
Output Voltage	DG_1_	9.743	0.031	3.85 1.03	1.65 0.15	1.52 1.77	0.51 0.17	0.08 0.02	≈0.0 ≈0.0
	DG_2_	60.972	19.972	≈0.0 1.45	2.47 0.37	1.67 1.96	0.57 0.37	0.09 0.08	≈0.0 ≈0.0
	DG_3_	9.534	0.0287	2.13 3.27	2.78 1.34	1.48 2.34	0.49 0.49	0.03 0.47	≈0.0 ≈0.0
	DG_4_	10.892	9.985	0.89 ≈0.0	0.51 1.73	1.29 5.92	0.31 0.52	≈0.0 1.89	≈0.0 ≈0.0
	DG_5_	1.842	0.0224	0.72 1.13	0.37 0.46	1.15 3.46	0.26 0.34	≈0.0 0.69	≈0.0 ≈0.0
	DG_6_	0.351	0.0169	0.19 0.78	0.03 0.23	1.07 1.78	0.19 0.24	≈0.0 0.35	≈0.0 ≈0.0

**Table 9:** Controller response parameters during concurrent 80% sensor effectiveness in DG_2_ and 90% sensor effectiveness in DG_4_ inside the designed range.

		% of Voltage Dip during Sensor Fault (%)		Control Response Parameters					
				% Overshoot (%)		Settling Time (s)		Steady State Error (%)	
		PI-Tuned	Proposed	PI-Tuned	Proposed	PI-Tuned	Proposed	PI-Tuned	Proposed
Output Voltage	DG_1_	8.729	≈0.0	0.4367	3.387	0.9219	0.6287	0.00781	≈0.0
	DG_2_	61.251	19.871	≈0.0	2.031	0.9512	0.6529	0.1034	≈0.0
	DG_3_	8.5625	≈0.0	0.4298	1.436	0.9037	0.6194	0.00651	≈0.0
	DG_4_	10.389	9.934	≈0.0	1.865	4.672	0.468	0.6621	≈0.0
	DG_5_	1.9761	≈0.0	0.311	0.461	3.473	0.211	0.1036	≈0.0
	DG_6_	0.8747	≈0.0	0.2984	0.207	1.267	0.1057	0.01453	≈0.0

**Table 10:** Controller response parameters during sensor effectiveness fault 90% in DG_5_.

		% of Voltage Dip during Sensor Fault (%)	Control Response Parameters		
			% Overshoot (%)	Settling Time (s)	Steady State Error (%)
		Proposed	Proposed	Proposed	Proposed
Output Voltage	DG_1_	Proposed ≈0.0 39.8734 ≈0.0 49.5439 ≈0.0 ≈0.0	Proposed 3.1046 2.2641 12.037 1.3474 2.0413 0.2281	Proposed 0.6387 0.7793 0.8367 0.8169 0.4536 0.1106	Proposed ≈0.0 ≈0.0 ≈0.0 ≈0.0 ≈0.0 ≈0.0
	DG_2_				
	DG_3_				
	DG_4_				
	DG_5_				
	DG_6_				

## Data Availability

Not applicable.

## Appendix A

The DC microgrid’s state equation matrices:

$$\begin{array}{c} A=\left[\begin{array}{ccc} \begin{array}{cc} 0.9951 & 0.03797\\ -0.01315 & 0.9046 \end{array} & \begin{array}{cc} 0.004429 & 6.703\times 10^{-5}\\ -2.971\times 10^{-5}\; & -3.045\times 10^{-7} \end{array} & \begin{array}{cc} 5.77\times 10^{-6} & 7.51\times 10^{-8}\\ -2.601\times 10^{-8} & -2.563\times 10^{-105}\; \end{array}\\ \begin{array}{cc} 0.004429 & 8.58\times 10^{-5}\\ -1.49\times 10^{-5} & -1.954\times 10^{-7} \end{array} & \begin{array}{cc} 0.998 & 0.0297\;\\ -0.006601\; & 0.9045 \end{array} & \begin{array}{cc} 0.002599 & 5.032\times 10^{-5}\;\\ -8.736\times 10^{-6} & -1.146\times 10^{-7} \end{array}\\ \begin{array}{c} \begin{array}{cc} 5.77\times 10^{-6} & 7.511\times 10^{-8}\\ -1.738\times 10^{-8} & -1.713\times 10^{-10} \end{array}\\ \begin{array}{cc} 5.131\times 10^{-9}\; & 5.033\times 10^{-11}\\ -1.743\times 10^{-11} & -1.376\times 10^{-13} \end{array}\\ \begin{array}{c} \begin{array}{cc} 3.563\times 10^{-12} & 2.805\times 10^{-14}\\ -4.869\times 10^{-15} & -3.209\times 10^{-17} \end{array}\\ \begin{array}{cc} 2.998\times 10^{-15} & 1.971\times 10^{-17}\\ -4.563\times 10^{-18} & -2.581\times 10^{-20} \end{array} \end{array} \end{array} & \begin{array}{c} \begin{array}{cc} 0.002599\; & 3.931\times 10^{-5}\\ -1.165\times 10^{-5} & -1.194\times 10^{-7} \end{array}\\ \begin{array}{cc} 3.466\times 10^{-6} & 3.523\times 10^{-8}\\ -1.562\times 10^{-8} & -1.202\times 10^{-10} \end{array}\\ \begin{array}{c} \begin{array}{cc} 3.208\times 10^{-9} & 2.458\times 10^{-11}\\ -5.463\times 10^{-12} & -3.37\times 10^{-14} \end{array}\\ \begin{array}{cc} 3.374\times 10^{-12} & 2.075\times 10^{-14}\\ -6.148\times 10^{-15} & -3.165\times 10^{-17} \end{array} \end{array} \end{array} & \begin{array}{c} \begin{array}{cc} 0.998\; & 0.03802\\ -0.008802 & 0.9044 \end{array}\\ \begin{array}{cc} 0.002662 & 5.153\times 10^{-5}\;\\ -1.785\times 10^{-5} & -2.341\times 10^{-7} \end{array}\\ \begin{array}{c} \begin{array}{cc} 3.696\times 10^{-6} & 4.809\times 10^{-8}8\\ -8.35\times 10^{-9} & -8.228\times 10^{-11} \end{array}\\ \begin{array}{cc} 5.181\times 10^{-95} & 5.082\times 10^{-11}\\ -1.176\times 10^{-11} & -9.29\times 10^{-14} \end{array} \end{array} \end{array} \end{array}\right.\\ \left.\begin{array}{ccc} \begin{array}{cc} 5.131\times 10^{-9} & 4.194\times 10^{-11}\\ -1.743\times 10^{-11}\; & -1.147\times 10^{-13} \end{array} & \begin{array}{cc} 3.563\times 10^{-12} & 3.896\times 10^{-14}\\ -9.712\times 10^{-15}\; & -8.889\times 10^{-17} \end{array} & \begin{array}{cc} 2.998\times 10^{-15}\; & 4.107\times 10^{-17}\\ -6.826\times 10^{-18} & -8.042\times 10^{-20} \end{array}\\ \begin{array}{cc} 3.466\times 10^{-6} & 3.758\times 10^{-8}\\ -7.83\times 10^{-9} & -6.429\times 10^{-11} \end{array} & \begin{array}{cc} 3.208\times 10^{-9} & 4.37\times 10^{-11}\\ -5.463\times 10^{-12} & -5.991\times 10^{-14} \end{array} & \begin{array}{cc} 3.374\times 10^{-12} & 5.533\times 10^{-14}\\ -4.611\times 10^{-15} & -6.33\times 10^{-17} \end{array}\\ \begin{array}{c} \begin{array}{cc} 0.002662 & 4.295\times 10^{-5}\\ -1.193\times 10^{-5} & -1.304\times 10^{-7} \end{array}\\ \begin{array}{cc} 0.9983 & 0.0317\\ -0.0131 & 0.9046 \end{array}\\ \begin{array}{c} \begin{array}{cc} 0.002772 & 4.472\times 10^{-5}\\ -9.318\times 10^{-6} & -1.019\times 10^{-7} \end{array}\\ \begin{array}{cc} 5.827\times 10^{-6} & 6.321\times 10^{-8}\\ -1.756\times 10^{-8} & -1.442\times 10^{-10} \end{array} \end{array} \end{array} & \begin{array}{c} \begin{array}{cc} 3.696\times 10^{-6} & 6.68\times 10^{-8}\\ -1.113\times 10^{-8} & -1.524\times 10^{-10} \end{array}\\ \begin{array}{cc} 0.002772 & 7.454\times 10^{-5}\\ -1.858\times 10^{-5} & -3.386\times 10^{-7} \end{array}\\ \begin{array}{c} \begin{array}{cc} 0.9971 & 0.05279\\ -0.006599 & 0.9047 \end{array}\\ \begin{array}{cc} 0.004192 & 0.0001128\\ -1.88\times 10^{-5} & -3.426\times 10^{-7} \end{array} \end{array} \end{array} & \begin{array}{c} \begin{array}{cc} 5.181\times 10^{-9} & 1.059\times 10^{-10}\\ -1.176\times 10^{-11} & -1.935\times 10^{-13}3 \end{array}\\ \begin{array}{cc} 5.827\times 10^{-6} & 1.58\times 10^{-7}\\ -2.626\times 10^{-8} & -5.393\times 10^{-10}0 \end{array}\\ \begin{array}{c} \begin{array}{cc} 0.004192 & 0.0001692\\ -1.41\times 10^{-5} & -3.854\times 10^{-7} \end{array}\\ \begin{array}{cc} 0.9947 & 0.07908\\ -0.008787 & 0.9042 \end{array} \end{array} \end{array} \end{array}\right] \end{array}$$

$$B=\left[\begin{array}{ccc} \begin{array}{c} \begin{array}{c} 0.0002676\\ 0.01318\\ 0.0 \end{array}\\ 0.0\\ \begin{array}{c} 0.0\\ 0.0\\ \begin{array}{c} 0.0\\ 0.0\\ \begin{array}{c} 0.0\\ 0.0\\ \begin{array}{c} 0.0\\ 0.0 \end{array} \end{array} \end{array} \end{array} \end{array} & \begin{array}{c} \begin{array}{c} 0.0\\ 0.0\\ 0.0001049 \end{array}\\ 0.006607\\ \begin{array}{c} 0.0\\ 0.0\\ \begin{array}{c} 0.0\\ 0.0\\ \begin{array}{c} 0.0\\ 0.0\\ \begin{array}{c} 0.0\\ 0.0 \end{array} \end{array} \end{array} \end{array} \end{array} & \begin{array}{ccc} \begin{array}{c} \begin{array}{c} 0.0\\ 0.0\\ 0.0 \end{array}\\ 0.0\\ \begin{array}{c} 0.000179\\ 0.00881\\ \begin{array}{c} 0.0\\ 0.0\\ \begin{array}{c} 0.0\\ 0.0\\ \begin{array}{c} 0.0\\ 0.0 \end{array} \end{array} \end{array} \end{array} \end{array} & \begin{array}{c} \begin{array}{c} 0.0\\ 0.0\\ 0.0 \end{array}\\ 0.0\\ \begin{array}{c} 0.0\\ 0.0\\ \begin{array}{c} 0.0002232\\ 0.01318\\ \begin{array}{c} 0.0\\ 0.0\\ \begin{array}{c} 0.0\\ 0.0 \end{array} \end{array} \end{array} \end{array} \end{array} & \begin{array}{cc} \begin{array}{c} \begin{array}{c} 0.0\\ 0.0\\ 0.0 \end{array}\\ 0.0\\ \begin{array}{c} 0.0\\ 0.0\\ \begin{array}{c} 0.0\\ 0.0\\ \begin{array}{c} 0.0001865\\ \;0.006608\\ \begin{array}{c} 0.0\\ 0.0 \end{array} \end{array} \end{array} \end{array} \end{array} & \begin{array}{c} \begin{array}{c} 0.0\\ 0.0\\ 0.0 \end{array}\\ 0.0\\ \begin{array}{c} 0.0\\ 0.0\\ \begin{array}{c} 0.0\\ 0.0\\ \begin{array}{c} 0.0\\ 0.0\\ \begin{array}{c} 0.0003726\\ 0.008809 \end{array} \end{array} \end{array} \end{array} \end{array} \end{array} \end{array} \end{array}\right]$$

*C* is a unit matrix.
